# Do Funders, Regulators, and Ethics Bodies Support Informative Trials? A Content Analysis of Global Guidance Documents

**DOI:** 10.1111/jep.70356

**Published:** 2026-01-22

**Authors:** Sarah R. Prowse, Miriam Brazzelli, Hanne Bruhn, Shaun Treweek

**Affiliations:** ^1^ Aberdeen Centre for Evaluation, University of Aberdeen Foresterhill Aberdeen UK

## Abstract

**Background:**

Trials are often poorly designed, address unimportant questions, or are conducted in ways that limit their usefulness. This contributes to research waste and undermines evidence‐based healthcare. We examined whether guidance from funders, regulators, and ethics review bodies supports the planning and approval of informative trials, with a focus on resources available to investigators and the role of oversight organizations.

**Methods:**

We conducted a directed qualitative content analysis of guidance documents from funders, regulators, and ethics review bodies across 13 countries and three multinational organizations. Documents were analysed using a coding framework based on the five conditions for trial informativeness identified by Zarin and colleagues: *Importance*, *Design*, *Feasibility*, *Integrity*, and *Reporting*.

**Results:**

The final content analysis contains 37 guiding documents, including 13 documents from funders, 14 from regulators, and 10 addressing the ethics of trials. While contextual examples varied, many of the recommended processes or actions to improve trial informativeness were consistent across global guidance from funders, regulators, and those with ethical oversight. The aspects of *Design* and *Integrity* were particularly well represented, whereas guidance on improving *Feasibility* was limited.

**Conclusion:**

The five key aspects of trial informativeness were reasonably described in some capacity across all forms of guidance, but may benefit from further examples or elaboration. Further research is needed to explore how trial guidelines and other supporting documents might incorporate more flexible, context‐sensitive approaches that reduce bureaucratic burden without compromising ethical and scientific rigour.

## 1 Introduction

Despite the good intentions of researchers and participants, uninformative trials are studies so poorly conceived or executed that they fail to fulfill their purpose of improving health outcomes [1, 2]. Clinical trial guidance documents issued by funders, regulators, ethics bodies, and other relevant stakeholders are essential to ensuring that trials are designed, conducted, and reported in ways that mitigate research waste. In the absence of guidance, there is potential for trials to be less informative, which raises ethical concerns and can diminish the overall impact of the research [1, 2].

Funders, regulators, and ethical agencies all seek to promote and uphold processes of clinical discovery that are both rigorous and useful [3]. However, the World Health Organization notes that while regulations and guidelines serve a well‐intended purpose within trial research, they can often lead to excessive bureaucracy with the ‘adverse consequence of reducing rather than improving the number of reliably informative trials across a range of settings’ [4]. There are also situational factors at the local, national, and international levels that further affect how guidance is understood and actioned in the context of wider universal scientific and ethical standards [4, 5].

As trials continue to innovate in their designs, so should the guidance provided by the bodies and agencies responsible for their funding, approval, and implementation [3]. The COVID‐19 pandemic highlighted the need for clarity in guiding documents to accelerate the development of effective therapies during a global health crisis and provided a compelling argument for guidelines that can adapt dynamically [6, 7, 8]. A better understanding of the current guidance provided to investigators, and the similarities and differences across agencies responsible for trials, can help to address wider challenges in improving trial informativeness.

Zarin and colleagues outlined five key attributes that define an *informative* trial: the trial should address a meaningful question (*Importance*), use appropriate and robust methods *(Design)*, be practically executable *(Feasibility)*, be conducted with scientific rigor *(Integrity)*, and share its findings transparently *(Reporting)* [1, 2]. As part of a broader initiative to enhance the informativeness of trials, encompassing a rapid review of global insights and interviews with trialists and other stakeholders, this content analysis of guiding trial documents aims to address the following research questions:

1. What is the current guidance provided to investigators to make trials informative?
2. What do organizations with legal responsibility for health and care trials and those that approve trials do to ensure trials are informative?

## 2 Method

This content analysis examines publicly available documents from organizations that fund, conduct, or approve clinical trials. Its primary aim is to better understand the guidance these documents provide to trial investigators and stakeholders to ensure that clinical trials are informative. A protocol was developed and is available on the Open Science Framework [9].

The content analysis focused on grey literature, specifically:

1. Guidance for investigators provided by funders.
2. Guidance, policy, or other documents intended to support organizations with legal responsibility for trials (i.e., sponsors), as well as those that approve trials (i.e., regulators, ethics bodies), in ensuring that trials are informative.

### 2.1 Sampling Strategy

The document sampling strategy included countries from across Africa, Asia, Australia/Oceania, Europe, North America, and South America (Table 1). Countries were selected on the following basis:

1. The number of trials actively being conducted across all trial phases, as reflected by a major trial registry (WHO International Clinical Trials Registry Platform [ICTRP], 2023−2024).
2. Consideration of any language constraints, primarily, was the document was available in English.
3. Preference from the study funder to consider areas of sub‐Saharan Africa.

**Table 1:** Countries and multinational organizations with guiding documents included in the content analysis.

Africa	Asia	Australia/Oceania	Europe	North America	South America	Multinational
Ethiopia	India	Australia	Germany	Canada	Brazil	EU Horizon
Kenya			Ireland	USA		Wellcome
Nigeria			Italy			WHO
South Africa			UK			

A selection of multinational bodies was also included to further reflect global guidelines. This approach was implemented to provide a broad enough dataset to yield meaningful insights, while ensuring data collection and analysis remained feasible.

### 2.2 Identification of Documents

We initially aimed to identify one primary funder for each country or multinational organization included (or more than one, in countries with a high volume of trials) based on information available through the WHO ICTRP for the period 2023−2024. Official websites were reviewed in the first instance to locate the main guidance used to support health‐related trials.

Once a funder or funding pathway was identified, we used the WHO ICTRP to search for related trials. For each trial, we aimed to identify the sponsor (if different from the funder), the ethics committee or ethical agency responsible for oversight, and the relevant regulatory authority. We then searched for and collected documents that were directly relevant to our research questions, prioritizing those clearly labeled as ‘guidance for applicants’ or policy and practice documents aimed at improving the informativeness of trials.

We set a limit of three key documents per funder, regulator, or ethics body. Documents were excluded if they were not directly relevant to the concept of trial informativeness, for example, forms or checklists solely related to administrative procedures or financial compliance. Decisions about the inclusion of documents were agreed by at least two members of the study team, with any disagreements resolved by a third team member. We did not aim for an equal distribution of documents across geographic regions or relevant guiding bodies, due to time and language constraints.

### 2.3 Analysis

The study employs a directed qualitative content analysis approach that builds on processes and actions identified by our team in a prior rapid review of global insights [10, 11]. This earlier review focused on Zarin et al. five key conditions for trial informativeness: *Importance, Design, Feasibility, Integrity, and Reporting*. From this rapid review, we derived 26 processes or actions associated with these five conditions, which served as our initial set of codes. This approach was used to identify conceptual patterns and relations across documents rather than to produce an itemised matrix of guidance elements, consistent with the aims of the analysis.

Deductive coding was used in the first instance for those elements that aligned with the rapid review, with further descriptive themes identified through inductive coding. A coding form was developed to record text already aligned with established insights, and to record further descriptive themes and supporting examples (Supporting Information S1: Material 1).

A record was also kept of the number of conditions for informativeness described in any capacity by each document using the following rubric, agreed by the study team:

- All conditions for informativeness are identified: Informativeness is comprehensively described with extensive detail and examples.
- 3–4 conditions for informativeness identified: Informativeness is reasonably described, but may benefit from further examples or elaboration.
- 1–2 conditions for informativeness identified: Informativeness is partially described with limited explanation.
- No conditions for informativeness identified: Informativeness is not described.

One member of the study team applied the coding framework across all identified documents. To ensure consistency, two additional study team members independently and blindly coded a 10% subset of the selected documents. Any disagreements or uncertainties during the coding process were resolved through consensus between reviewers. The multidisciplinary study team included researchers with backgrounds in clinical trial design and methodology, evidence synthesis, and information science, with prior experience in qualitative methods and content analysis. A completed Standards for Reporting Qualitative Research (SRQR) checklist is provided to support transparency and completeness in reporting (Supporting Information S1: Material 2).

## 3 Results

**Figure 1:**
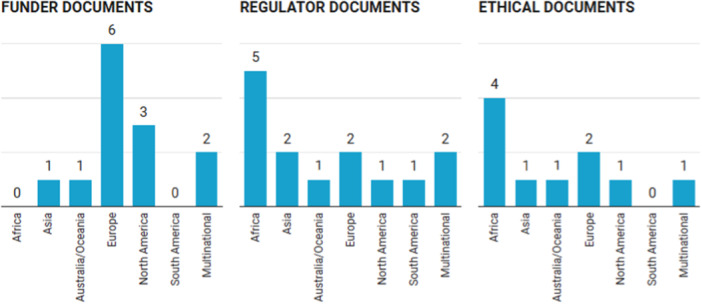
Document collection summary by region.

The content analysis included 37 documents across 13 countries and three wider multinational agencies. Of these, 13 documents were sourced from funders, 14 from regulators, and 10 addressed the ethical conduct of trials. Figure 1 provides a summary of document distribution by geographic region. A complete bibliography detailing the documents by type and geographic region is provided in Supporting Information S1: Material 3. Only one document was not available in English and was translated by a member of the study team in accordance with the research protocol [9, 12]. Results are presented in an integrated format to avoid duplication and to support cross‐organisational comparison of guidance from funders, regulators, and ethics bodies (Table 2). This structure reflects the directed content analysis approach, which emphasises conceptual synthesis across sources rather than document‐by‐document reporting.

**Table 2:** Guidance on informativeness provided by funders, regulatory authorities, and ethics bodies; key differences between supporting examples are highlighted in bold. Gray boxes reflect instances where guidance on the process or action to improve informativeness was either not found in the organization's materials or was not evident in our analysis.

Recommended process or action to improve informativeness:	Supporting examples from FUNDER documents:	Supporting examples from REGULATOR documents:	Supporting examples from ETHICS documents:
***1. Importance: trial hypothesis is likely to inform an importance scientific, medical, or policy decision***			
Justify the need for the proposed trial including anticipated impacts to health and wider society	Evidence to justify the need for the proposed trial and its anticipated broader impacts can include information in the literature, **knowledge of biological mechanisms, preliminary data**, and pre‐clinical and/or clinical studies [13, 14, 15, 16, 17, 18]	Evidence to justify the need for the proposed trial and its anticipated broader impacts can include information in the literature **or public domain**, and pre‐clinical and/or clinical studies; **global capacity for clinical trial research can be strengthened when multiple stakeholders explore opportunities to coordinate research priorities** [4, 19, 20, 21, 22]	Evidence to justify the need for the proposed trial and its anticipated broader impacts can include information in the literature **or public domain**, and pre‐clinical and/or clinical studies; **systematic reviews and meta‐analyses should adhere to standards such as the Preferred Reporting Items for Systematic Reviews and Meta‐Analyses (PRISMA)** [23, 24, 25, 26, 27, 28, 29]
Conduct a search of global clinical trial databases to avoid duplication of research effort	The proposed trial should only replicate existing research when that is the explicit aim [13]; any overlaps identified through a search of global databases should be clearly justified in the context of advancing scientific understanding [13, 16]		
Include patients as partners at the outset of the trial development process	Emphasizing the role of patients in guiding the development of trial hypotheses can better address issues important to patients, and the proposed intervention is more likely to be seen by patients as acceptable [13, 14, 15, 16, 30]	Emphasizing the role of patients in guiding the development of trial hypotheses can better address issues important to patients, and the proposed intervention is more likely to be seen by patients as acceptable [4, 22, 31]	Emphasizing the role of patients in guiding the development of trial hypotheses can better address issues important to patients, and the proposed intervention is more likely to be seen by patients as acceptable [23, 27, 28, 32, 33]
Extend and formalise traditional research practices through Open Science (OS)	Pre‐registering prospective studies on online platforms or submitting a registered report that specifies hypotheses, study design, and analysis plans prior to data collection can enhance research transparency, regardless of peer review status [13, 18, 30, 34, 35]	Pre‐registering prospective studies on online platforms or submitting a registered report that specifies hypotheses, study design, and analysis plans prior to data collection can enhance research transparency, regardless of peer review status [4, 22]	Pre‐registering prospective studies on online platforms or submitting a registered report that specifies hypotheses, study design, and analysis plans prior to data collection can enhance research transparency, regardless of peer review status [25, 33]
***2. Design: trial methods are likely to provide meaningful evidence related to the study hypothesis***			
Select fit‐for‐purpose tools to further support a process of informed trial design	Trial design tools can be used to plan, structure, and optimise the design of a trial before it begins; **cited examples include the *Core Outcome Measures in Effectiveness Trials (COMET) Initiative*** [36], ***Standard Protocol Items: Recommendations for Interventional Trials (SPIRIT)*** [37], ***Enhancing the QUAlity and Transparency Of health Research (EQUATOR) Network*** [38], **and the *Consolidated Standards of Reporting Trials (CONSORT)* statement** [13, 16, 39]	Trial design tools can be used to plan, structure, and optimise the design of a trial before it begins; **cited examples include the *Standard Protocol Items: Recommendations for Interventional Trials (SPIRIT)*** [37] **checklist and *WHO Global Benchmarking Tool*** [40] **for evaluation of national regulator systems for medicinal products** [4, 41]	
Accountability from trial funders and/or sponsors to ensure trial design considerations are appropriate and adequately justified	Trial funders and/or sponsors should justify the selection and validation of all trial design elements, including the trial objectives, inclusion and exclusion criteria (including equity, diversity, and inclusion considerations), interventions and comparators, sample size, outcomes, feasibility, and suggested trial analysis and follow‐up [13, 14, 15, 16, 17, 18, 30, 42]	Trial funders and/or sponsors should justify the selection and validation of all trial design elements, including the trial objectives, inclusion and exclusion criteria (including equity, diversity, and inclusion considerations), interventions and comparators, sample size, outcomes, feasibility, and suggested trial analysis and follow‐up [4, 19, 20, 21, 22, 31, 41, 43, 44, 45, 46]	Trial funders and/or sponsors should justify the selection and validation of all trial design elements, including the trial objectives, inclusion and exclusion criteria (including equity, diversity, and inclusion considerations), interventions and comparators, sample size, outcomes, feasibility, and suggested trial analysis and follow‐up [23, 24, 26, 27, 28, 32, 33, 47]
Further deliberation of critical ethical issues within trial protocol development	Substantive discussion of specific ethical issues (e.g., participant autonomy and protection) is rarely included in clinical trial protocols; protocols should demonstrate consideration for jurisdictional and global standards of ethical research while critically assessing and justifying specific design considerations [13, 16, 30] (*see Integrity)*	Substantive discussion of specific ethical issues (e.g., participant autonomy and protection) is rarely included in clinical trial protocols; protocols should demonstrate consideration for jurisdictional and global standards of ethical research while critically assessing and justifying specific design considerations [19, 20, 21, 41, 43, 44, 45, 46] (*see Integrity)*	Substantive discussion of specific ethical issues (e.g., participant autonomy and protection) is rarely included in clinical trial protocols; protocols should demonstrate consideration for jurisdictional and global standards of ethical research while critically assessing and justifying specific design considerations [23, 24, 27, 32, 33] (*see Integrity)*
*Risk‐benefit analysis and participant vulnerability*			Not all trials are considered high risk; however, some trials may involve the risk of serious harm or death which should be justified appropriately in the protocol, particularly for trials that may involve participants whose circumstances make them vulnerable in the context of the research [23, 24, 26, 28]
*Informed consent process*			Trial may employ a range of methodological approaches to informed consent; however, the protocol should demonstrate a continuous process that includes providing relevant information to potential participants, ensuring their competence, presenting information in an easily comprehensible manner, and assuring the voluntariness of participation [23, 24, 26, 28, 32]
*Consideration of prior scientific review*			Trial designs that have undergone scientific review may be methodologically sound but still unethical; ethical governing bodies may disagree with prior reviews, and request amendments to the trial design to better reflect participant autonomy and protection [26, 27]
Integrate a patient‐centric approach throughout the trial design process (e.g., weighing participant considerations such as convenience, risk‐to‐benefit ratio, social interaction, partnership, and altruism)	Consideration of patient needs, notably in early phase trial development, can lead to fewer protocol amendments, improved endpoints, improved feasibility (recruitment and retention), and higher patient satisfaction; a patient‐centric approach will vary by jurisdiction and should consider the trial environment, including how trials conducted in low‐ and middle‐income countries (LMICs) may differ from high income (HMIC) contexts [13, 16, 35]	Consideration of patient needs, notably in early phase trial development, can lead to fewer protocol amendments, improved endpoints, improved feasibility (recruitment and retention), and higher patient satisfaction; a patient‐centric approach will vary by jurisdiction and should consider the trial environment, including how trials conducted in low‐ and middle‐income countries (LMICs) may differ from high income (HMIC) contexts [22, 31, 45]	Consideration of patient needs, notably in early phase trial development, can lead to fewer protocol amendments, improved endpoints, improved feasibility (recruitment and retention), and higher patient satisfaction; a patient‐centric approach will vary by jurisdiction and should consider the trial environment, including how trials conducted in low‐ and middle‐income countries (LMICs) may differ from high income (HMIC) contexts [23, 32, 33, 48]
Consider a diversity of expertise within trial teams to ensure conditions for informativeness are incorporated throughout all aspects of trial planning and execution	Expertise should be sought across all areas of trial design and development, including information technology, data development, scientific protocol development, clinical affairs, and financial operations; diversity is notably relevant to multicentre trials where expertise may span multiple teams working to achieve a shared outcome [16, 17, 18, 34, 35, 49]	Expertise should be sought across all areas of trial design and development, including information technology, data development, scientific protocol development, clinical affairs, and financial operations; diversity is notably relevant to multicentre trials where expertise may span multiple teams working to achieve a shared outcome [4, 50]	Expertise should be sought across all areas of trial design and development, including information technology, data development, scientific protocol development, clinical affairs, and financial operations; diversity is notably relevant to multicentre trials where expertise may span multiple teams working to achieve a shared outcome [23, 25, 26, 27, 28]
Conduct a pre‐submission consultation meeting with relevant regulatory authorities		A pre‐submission meeting creates an opportunity for the trial sponsor and/or funder and the regular to deliberate on the study plan and address any areas of uncertainty prior to submission of a formal application [41, 44, 50, 51]	
Implement a ‘quality‐by‐design’ approach to clinical trials		A ‘quality‐by‐design’ approach enables organizations to prioritise the most critical determinants of a trial's quality, identify non‐essential activities that can be eliminated to streamline trial conduct and oversight, and formulate appropriate plans to define, avoid, mitigate, monitor, and address important errors [4, 19, 20, 41, 45]	A ‘quality‐by‐design’ approach enables organizations to prioritise the most critical determinants of a trial's quality, identify non‐essential activities that can be eliminated to streamline trial conduct and oversight, and formulate appropriate plans to define, avoid, mitigate, monitor, and address important errors [25]
Establish research forums and other collaborative networks as a source of trial design feedback and support		Research forums and other collaborative networks can provide useful feedback when considering trial design and conduct, such as obtaining approvals, data management, and developing good work relationships with funders; low‐resource settings may also uniquely benefit from further collaboration when considering trial design and implementation [4, 22]	
***3. Feasibility: the trial must be demonstrably feasible (e.g., it must have a realistic plan for recruiting sufficient participants)***			
If appropriate, consider a pilot or feasibility study to avoid research waste and de‐risk funding investment(s)	Pilot or feasibility studies are potentially useful in assessing the efficacy of a method and whether a more expensive, large‐scale trial is merited [14, 15, 49]		
Implement a formal process of recruitment monitoring across the study timeline	Under‐recruitment can be addressed through reporting at regular intervals and allows for consideration of additional recruitment strategies in the event of a shortfall [17, 42]		
Integrate qualitative evidence when assessing the feasibility of a trial		Qualitative research findings can further explore lived experiences of a disease condition, such as what constitutes normal care, and other barriers and enablers to participation within a trial [41]	Qualitative research findings can further explore lived experiences of a disease condition, such as what constitutes normal care, and other barriers and enablers to participation within a trial [27]
Include community members in participant recruitment and retention strategies		**Concepts of community will vary by global context, but may be particularly relevant in low‐resource settings when considering trial feasibility**; community members can provide valuable feedback within retention strategies, such as best practices for communication with potential trial participants [4, 31]	**The recruitment strategy should be relevant to the trial methodology and detailed in the trial protocol;** community members can provide valuable feedback within retention strategies, such as best practices for communication with potential trial participants [23, 27, 28, 33]
Utilise the knowledge of local health professionals to better inform feasibility or pilot studies		Integration of local health professionals within the trial process can further contextualize issues of recruitment and retention, and help to ensure that the required sample size can be reached [4, 22]	
Consider targeted engagement and inclusion strategies for potentially vulnerable participants		Ethical and practical considerations differ when recruiting minors, individuals lacking capacity to consent, individuals in dependent relationships, and others; the health needs and priorities of potentially vulnerable trial participants should be considered, if applicable, within recruitment strategies [45]	
***4. Integrity: the trial must be conducted and analysed in a scientifically valid manner that is faithful to the design***			
Conduct a process of ethical review and, if applicable, the establishment of an independent ethics review board	Ethical review processes will differ across jurisdictions; for multi‐site trials, teams should aim to streamline and harmonize ethics submissions where possible to minimize duplication of effort and ensure clarity in trial processes [13, 34, 35]	Ethical review processes will differ across jurisdictions; for multi‐site trials, teams should aim to streamline and harmonize ethics submissions where possible to minimize duplication of effort and ensure clarity in trial processes [4, 22, 44, 45, 50]	Ethical review processes will differ across jurisdictions; for multi‐site trials, teams should aim to streamline and harmonize ethics submissions where possible to minimize duplication of effort and ensure clarity in trial processes [23, 24, 25, 26, 27, 28, 32, 33, 47, 48]
Compliance with all applicable legislation, regulations, guidelines and international best practices with an emphasis on the rights and safety of trial participants	Global best practice documents for the conduct of trials include (among others) the Declaration of Helsinki, the Belmont Report, Nuremberg Code, and guidelines from the International Council for Harmonisation including Good Clinical Practice (GCP) to ensure ethical and scientific quality [12, 16, 30, 34, 35, 49]	Global best practice documents for the conduct of trials include (among others) the Declaration of Helsinki, the Belmont Report, Nuremberg Code, and guidelines from the International Council for Harmonisation including Good Clinical Practice (GCP) to ensure ethical and scientific quality [20, 41, 44, 45, 50]	Global best practice documents for the conduct of trials include (among others) the Declaration of Helsinki, the Belmont Report, Nuremberg Code, and guidelines from the International Council for Harmonisation including Good Clinical Practice (GCP) to ensure ethical and scientific quality [24, 25, 28, 33]
Establish a trial steering committee to provide independent oversight for the trial	A trial steering committee ensures the trial is conducted in accordance with applicable legislation, regulations, guidelines, and best practices and can advise or take further action on potential research integrity violations [12, 16, 34, 35, 49]	A trial steering committee ensures the trial is conducted in accordance with applicable legislation, regulations, guidelines, and best practices and can advise or take further action on potential research integrity violations [45]	A trial steering committee ensures the trial is conducted in accordance with applicable legislation, regulations, guidelines, and best practices and can advise or take further action on potential research integrity violations [25]
Establish an independent committee for data protection and monitoring	A data protection committee oversees compliance with relevant data protection laws and best practices to ensure robust data handling; multi‐site trials may have differing jurisdictional data considerations, or unique contextual factors such as the use of Indigenous data and knowledge [13, 16, 17, 34, 49]	A data protection committee oversees compliance with relevant data protection laws and best practices to ensure robust data handling; multi‐site trials may have differing jurisdictional data considerations, or unique contextual factors such as the use of Indigenous data and knowledge [19, 20, 22, 41, 45]	A data protection committee oversees compliance with relevant data protection laws and best practices to ensure robust data handling; multi‐site trials may have differing jurisdictional data considerations, or unique contextual factors such as the use of Indigenous data and knowledge [23, 24, 25, 28]
Clearly define roles and responsibilities within a trial team (e.g., Principal Investigator, study co‐ordinator, data manager, research integrity officer, etc.)	The roles of the trial team should clearly define responsibility for specific aspects of the trial, such as general management, medical care of participants, safety reporting and compliance, data handling and record keeping, among others [16, 35]	The roles of the trial team should clearly define responsibility for specific aspects of the trial, such as general management, medical care of participants, safety reporting and compliance, data handling and record keeping, among others [43, 52]	The roles of the trial team should clearly define responsibility for specific aspects of the trial, such as general management, medical care of participants, safety reporting and compliance, data handling and record keeping, among others [25, 26]
Further development of skills‐based training to ensure the quality conduct of trials	Trial teams may benefit from training beyond standard good practice guidelines in obtaining informed consent, ensuring protocol compliance, and protecting participants' health and safety; **mentorship schemes can support professional development and the ability to apply knowledge and skills appropriately in the context of trial research** [13, 18, 35, 53]	Trial teams may benefit from training beyond standard good practice guidelines in obtaining informed consent, ensuring protocol compliance, and protecting participants' health and safety [19, 22, 41, 45, 51]	Trial teams may benefit from training beyond standard good practice guidelines in obtaining informed consent, ensuring protocol compliance, and protecting participants' health and safety; **mentorship schemes can support professional development and the ability to apply knowledge and skills appropriately in the context of trial research** [25, 28, 32, 33, 48]
Establish a system for structured, participant‐centred communication during the trial		Given the resources available, trial teams should ensure timely, relevant updates are delivered to participants as established in the protocol to maintain informed consent and engagement throughout the trial including changes to risk or design assumptions [4, 46]	
Ensure adequate trial site facilities		The trial site must have appropriate facilities to conduct the protocol including physical structure, equipment/tools, and human resources; first‐in‐human trials should take place in appropriate clinical facilities by staff with an appropriate level of training including a designated pharmacy [41, 52]	
Post‐trial obligations	Trial teams have an ethical obligation to conduct any post‐research health monitoring related to trial participation as specified in the trial protocol [18]		
***5. Reporting: systems are in place to ensure timely, complete, and accurate reporting***			
Mandatory requirement to proactively register trials alongside a supporting system of monitoring to ensure compliance with registration, up‐to‐date record keeping, and timely publication of results	Publicly specifying details on trial methodology and conduct before enrolling participants increases transparency, decreases selective reporting and subsequent publication bias, and ensures an ethical responsibility to publicly report trial results [30, 34, 35]	Publicly specifying details on trial methodology and conduct before enrolling participants increases transparency, decreases selective reporting and subsequent publication bias, and ensures an ethical responsibility to publicly report trial results [4, 22, 41, 43, 45, 52]	Publicly specifying details on trial methodology and conduct before enrolling participants increases transparency, decreases selective reporting and subsequent publication bias, and ensures an ethical responsibility to publicly report trial results [23, 24, 32, 33]
Dissemination of trial outputs and wider knowledge mobilization	Trial findings should be shared through accessible, inclusive formats with relevant audiences as appropriate, including the trial participants; broader knowledge mobilization should promote the uptake of results into policy and practice at local, national, and/or international levels [13, 16, 30, 49]	Trial findings should be shared through accessible, inclusive formats with relevant audiences as appropriate, including the trial participants; broader knowledge mobilization should promote the uptake of results into policy and practice at local, national, and/or international levels [4, 20, 22, 43, 46]	Trial findings should be shared through accessible, inclusive formats with relevant audiences as appropriate, including the trial participants; broader knowledge mobilization should promote the uptake of results into policy and practice at local, national, and/or international levels [26, 27, 28, 32, 33]
Adherence to best publication practices for the results of trials	**The International Committee of Medical Journal Editors (ICMJE) and Committee on Publication Ethics (COPE) provide guidelines for researchers on publication ethics, research integrity, and authorship to avoid misconduct; researchers should consider the use of anti‐plagiarism software to ensure the originality of findings for publication** [35]		**Researchers should consider the quantity and quality of trial outputs to best enable readers to understand the full context of the research; in addition to publications in books and journals, researchers can also consider other platforms, such as academic or data repositories, so long as alternatives have established guidelines on good research practice** [25]
Maintenance of trial records for further reporting purposes	Research records should be maintained after trial completion as required by the governing authority to enable potential verification, re‐use, and oversight of potentially sensitive data [35]		Research records should be maintained after trial completion as required by the governing authority to enable potential verification, re‐use, and oversight of potentially sensitive data [23]

### 3.1 Guidance on Informativeness Provided by **Funders**

Within funder documents, the most described conditions for informativeness were *Design* and *Integrity*, both found in 85% of documents. *Importance* was described by 65% of documents, followed by *Reporting* in 54% of documents. *Feasibility* was the least described condition for informativeness, found in only 38% of documents. On average, approximately three conditions for informativeness were described across funder documents, suggesting informativeness overall was reasonably described but may benefit from further examples or elaboration.

A total of 22 recommended processes or actions to improve informativeness were noted within the guiding documents provided by funders, as summarized in Table 2. One third of the recommendations address *Integrity* (7/22, 32%). Unlike documents from regulators and ethics bodies, most funder documents were sourced from Europe and North America (9/13, 69%).

### 3.2 Guidance on Informativeness Provided by **Regulatory Authorities**

The most frequently described condition for informativeness provided by regulators was *Design*, mentioned in 93% of the documents reviewed. This was followed by *Integrity* (86%), *Reporting* (57%), *Importance* (43%), and *Feasibility* (36%). On average, regulatory documents addressed approximately three conditions for informativeness, suggesting informativeness overall was reasonably described but may benefit from further examples or elaboration.

In total, 25 recommended processes or actions aimed at enhancing informativeness were identified across the guidance documents, as summarized in Table 2. Both *Design* and *Integrity* had 8 recommendations, accounting for approximately 64% of the suggested processes or actions to improve trial informativeness. Geographically, most documents were sourced from Africa (6/14, 43%).

### 3.3 Guidance on Informativeness Provided by **Ethics Bodies**

The most frequently described condition for informativeness in documents from ethics bodies was *Design*, with all documents (100%) providing some guidance on trial methodologies. Ethics guidance documents also commonly addressed *Importance*, *Integrity*, and *Reporting*, each mentioned in 80% of the documents. *Feasibility* was again the least described condition for informativeness, with only 40% of ethics documents providing relevant guidance. On average, approximately four conditions for informativeness were described across the documents, suggesting informativeness overall was reasonably described but may benefit from further examples or elaboration.

A total of 20 recommended processes or actions to improve informativeness were identified across guidance documents provided by ethics bodies, as summarized in Table 2. A distinguishing feature of ethics guidance was the specificity of recommendations, including consistently well‐developed guidance for trial protocols, risk‐benefit assessments, the protection of vulnerable populations, the informed consent process, and consideration of prior scientific review. Ethics documents were most likely to recommend processes or actions related to *Integrity* (6/20, 30%) and *Design* (5/20, 25%). Most of the ethical documents were sourced from Africa (40%).

### 3.4 Shared Messaging Across Funders, Regulators, and Ethics Bodies

While contextual examples vary, many of the recommended processes or actions to improve trial informativeness are consistent across guidance from funders, regulators, and ethics bodies. *Feasibility* stands out as the least addressed condition across all three document types. Table 3 summarises the shared messaging across funders, regulators, and ethics bodies for each of the five conditions of trial informativeness.

**Table 3:** Shared messaging across funders, regulators, and ethics bodies; *only reflected in regulator and ethics guidance documents, as Feasibility was the least addressed condition for informativeness throughout the content analysis.

1. **Importance: trial hypothesis is likely to inform an important scientific, medical, or policy decision**
Justify the need for the proposed trial, including anticipated impacts on health and wider society Include patients as partners at the outset of the trial development process Extend and formalise traditional research practices through Open Science (OS)
2. **Design: trial methods are likely to provide meaningful evidence related to the study hypothesis**
Accountability from trial funders and/or sponsors to ensure trial design considerations are appropriate and adequately justified Further deliberation of critical ethical issues within trial protocol development Integrate a patient‐centric approach throughout the trial design process Consider a diversity of expertise within trial teams to ensure conditions for informativeness are incorporated throughout all aspects of trial planning and execution
3. **Feasibility: the trial must be demonstrably feasible (e.g., it must have a realistic plan for recruiting sufficient participants)**
Integrate qualitative evidence when assessing the feasibility of a trial* Include community members in participant recruitment and retention strategies*
4. **Integrity: the trial must be conducted and analysed in a scientifically valid manner that is faithful to the design**
Conduct a process of ethical review and, if applicable, the establishment of an independent ethics review board Compliance with all applicable legislation, regulations, guidelines, and international best practices with an emphasis on the rights and safety of trial participants Establish a trial steering committee to provide independent oversight for the trial Establish an independent committee for data protection and monitoring Clearly define roles and responsibilities within a trial team (e.g., Principal Investigator, study co‐ordinator, data manager, research integrity officer, etc.) Further development of skills‐based training to ensure the quality conduct of trials
5. **Reporting: systems are in place to ensure timely, complete, and accurate reporting**
Mandatory requirement to proactively register trials alongside a supporting system of monitoring to ensure compliance with registration, up‐to‐date record keeping, and timely publication of results Dissemination of trial outputs and wider knowledge mobilization

## 4 Discussion

This content analysis provides important insights into how current guidance supports trial informativeness and how funders, regulators, and ethics bodies converge and diverge in their recommendations. Using Zarin et al. five conditions for informative trials (*Importance*, *Design*, *Feasibility*, *Integrity*, and *Reporting*), we identified consistent areas of strength, as well as gaps, across the documents reviewed.

*Importance* was commonly emphasized across all document types, particularly the need to justify the relevance of the research question and the anticipated contribution to scientific knowledge, as well as the broader impact on both health and society [4, 13, 14, 15, 16, 17, 18, 19, 20, 21, 22, 23, 24, 25, 26, 27, 28]. This aligns with the World Health Organization's broader position that the global ‘trial ecosystem’ benefits when multiple stakeholders explore opportunities to coordinate research priorities [4, 22]. Enhancing transparency through Open Science practices can accelerate innovation in trials research through widespread sharing of information while avoiding duplication of effort and mitigating subsequent research waste [4, 19, 20, 21, 22]. However, while these principles are widely endorsed, the degree to which they are operationalised varies in practice. Empirical research has, for example, identified variability in how funders and ethics committees require or enforce the use of systematic reviews in trial justification, with responsibility for assessing prior evidence often left to investigators or peer reviewers [54].

Much of the guidance provided by funders, regulators, and ethics bodies focused on the conditions of *Design* and *Integrity* to enhance trial informativeness, which were often interlinked, as the latter indicates that the trial must be conducted and analyzed in a scientifically valid manner that is faithful to the design [2]. Regulatory bodies, whose mandates include ensuring that trials meet safety, efficacy, and scientific integrity standards, understandably provided detailed guidance in these areas [55]. However, over‐interpretation of regulatory guidance can lead to excessive trial monitoring and costly protocol amendments that ultimately reduce patient recruitment and retention without clear evidence of improved trial quality [56, 57, 58]. These issues are especially pronounced in multisite trials where regulatory guidance differs between jurisdictions, and in low‐resource settings where the practicalities of actioning varying regulatory requirements can be challenging [4, 59].

One practical solution offered in regulatory guidance documents is to conduct a pre‐submission or pre‐application meeting. The National Agency for Food and Drug Administration and Control of Nigeria describes this optional meeting as ‘an opportunity for the Sponsor and the Regulator to deliberate on the potential study plan, to address some grey areas, prior to submission of the clinical trial application’ [51]. Similar guidance was found in documents from India, Ireland, and Kenya [41, 44, 50]. Pre‐submission meetings have been shown to promote dialogue between sponsors, regulators, and when feasible, ethics reviewers. Evidence suggests that they can expedite approvals by allowing the opportunity to request clarification on submission guidance [55]. All forms of trials, regardless of context or setting, could benefit from a pre‐submission meeting as the approval process can be time‐consuming and costly, and poorly designed and executed trials often lead to delays in generating high‐quality evidence [60].

A recurring theme in guidance documents related to *Design* and *Integrity* was streamlining processes or actions to improve trial informativeness, such as the use of tools to plan, structure, and optimise the design of a trial. Funders cited specific examples of design tools such as COMET, SPIRIT, CONSORT, and the EQUATOR network [13, 16, 36, 37, 38, 39]. These resources can reduce research waste and improve informativeness by enabling trial teams to consider differing aspects and contexts of trial design, including ethical issues within complex and evolving research environments [9]. Guidance from funders also encouraged harmonization of ethics review processes in multisite or global trials, to further minimize duplication of effort and avoid redundancy, and ensure clarity in trial processes [13, 34, 35]. However, this is not always practically feasible. For example, the United States currently dominates the global trial market but does not always benefit from a centralized (national) ethics approval process [61]. Instead, Institutional Review Boards (IRBs) found within agencies that conduct trials may provide an independent review of protocols to determine whether they meet ethical standards [62, 63]. As each IRB may work to its own schedule with differing processes and responses to a protocol, the start of a multisite trial is only as fast as the slowest IRB [62].

Ethics bodies provided, in some capacity, detailed guidance on *Design* elements such as risk‐benefit analysis, informed consent, participant vulnerability, and consideration of prior scientific review [23, 24, 26, 27, 28, 32]. These aspects ensure not only participant autonomy but can further support trials that are equitable, diverse, and inclusive. When the trial design is not carefully considered, the results ‘may be less generalizable to groups who could potentially benefit from the findings […] sometimes most affecting the groups with the highest burden from a particular disease or condition’ [4]. The importance of ethical review and the establishment of review boards or committees, if needed, was also uniformly highlighted across documents from funders and regulators. Further, trial steering and data committees were also consistently recommended across all three types of guidance documents to uphold ethical standards throughout the conduct of a trial.

The condition for trial informativeness least addressed across all document types was *Feasibility*, which was mentioned in 38% of funder documents, 36% of regulator documents, and 40% of ethics‐related documents. Zarin and colleagues described *Feasibility* as the presence of a realistic plan to recruit and retain a sufficient number of participants. Both regulators and ethicists emphasised the value of involving community members in the development of recruitment and retention strategies [4, 23, 27, 28, 31, 33]. Concepts of ‘community’ will vary by global context and may be especially critical in low‐resource settings when considering trial feasibility. For instance, the South African National Health Research Council states: ‘Sometimes a community is identified geographically, other times, it is identified by association or age or other social determinant […] engagement always requires a level of humility on the part of the researchers as the community is the expert and holder of local knowledge’ [28]. This local expertise can support trial teams in developing best practices for communication with potential participants and designing retention strategies that are aligned with community norms and values [4, 23, 27, 28, 31, 33].

The aspect of *Reporting* across all document types emphasised the importance of proactively registering trials in an appropriate trial registry. Such registration enhances research transparency by reducing selective reporting and subsequent publication bias [9]. It also supports *Integrity* by adhering to established ethical standards, such as the Declaration of Helsinki, which states: ‘Researchers have a duty to make publicly available the results of their research on human participants and are accountable for the timeliness, completeness, and accuracy of their reports’ [64]. In addition, dissemination of trial outputs and how these outputs are mobilized into wider health and societal benefits was also highlighted throughout the guidance. Knowledge mobilization was described as a communicative process where trial outputs should be shared with participants and relevant communities to further translate complex findings into real‐world solutions [13]. While approaches will vary by context, a core aim of knowledge mobilization is to bridge the gap between new evidence generated by trials and how that evidence informs practice and policy [13, 27, 30]. This also reflects *Importance*, specifically the need to assess from the outset the potential broader impacts of a trial on health and society.

Although not specifically reviewed for this content analysis, the ICH guideline ICH E8, *General considerations for clinical studies*, was frequently referenced across guidance documents as a key resource that should be consulted alongside regional advice [65]. A similar role was noted for other ICH documents, such as ICH E6, *Good clinical practice* [66]. While these documents provide internationally recognized ethical and scientific quality standards for trials, they have also been subject to criticism, particularly regarding their interpretation and practical implications. Previous research suggests that ICH E8 could be strengthened by more explicitly promoting a proactive, risk‐based approach to trial design and conduct. Meanwhile, ongoing revisions to ICH E6 aim to reflect the views of trial stakeholders by incorporating more flexible processes and simplified requirements to better support contemporary research practices [67, 68, 69]. These developments echo earlier observations made in the context of the COVID‐19 pandemic, which highlighted the importance of adaptive, responsive trial guidance in a rapidly evolving research landscape [6, 7, 8].

This content analysis presented some limitations, which are worth noting. We did not aim for an equal distribution of documents across regions, primarily due to language constraints encountered during the search process. Moreover, by analyzing documents from funders, regulators, and ethics bodies collectively, it was not feasible to conduct a more granular assessment of the specific processes and actions recommended to enhance trial informativeness. The recommendations presented for each type of document, along with reflections on shared messaging across all three types, are intended to offer a broad overview of both unique and overlapping perspectives on informativeness. The level of detail in describing processes or recommended actions varied widely; some documents provided rich contextual information, while others offered minimal elaboration. This made our effort to summarise elements across documents challenging. To illustrate how trial informativeness can be implemented across different contexts, we have included examples from guidance documents alongside the recommended processes or actions. In our discussion, we have also noted where certain aspects of existing guidance may face practical limitations in real‐world application.

This study forms part of a broader programme of work examining how trial informativeness is conceptualised and operationalised across the clinical research ecosystem. This programme includes our published rapid review, the present content analysis, and forthcoming qualitative interviews with individuals involved in the funding, oversight, and conduct of trials. The next stage of this work is a mapping exercise that will build on and extend the present synthesis by combining these complementary data sources to provide a more comprehensive understanding of how guidance on trial informativeness is articulated and applied across differing contexts.

## 5 Conclusion

Overall, the five key aspects that can make a trial more informative were reasonably described in some capacity across guidance from funders, regulators, and ethics bodies, but may benefit from further examples or elaboration. The aspects of *Design* and *Integrity* were particularly well represented, whereas guidance on improving *Feasibility* was limited, highlighting an important area for greater focus. A consistent emphasis was placed on the need for collaboration across all jurisdictional levels, including the involvement of patients and relevant communities in shaping both the successful conduct and broader impact of trials. While such guiding documents serve an important and warranted purpose, further research is needed to explore how guidelines might incorporate more flexible, context‐sensitive approaches that reduce bureaucratic burden without compromising ethical and scientific rigour. Doing so would provide clearer, more actionable support for trial teams seeking to enhance informativeness, while aligning with the dynamic and evolving landscape of global clinical research.

## Author Contributions

**Sarah R. Prowse:** conceptualization, methodology, formal analysis, investigation, resources, writing − original draft, project administration. **Miriam Brazzelli:** conceptualization, methodology, validation, writing – review and editing, supervision. **Hanne Bruhn:** conceptualization, methodology. **Shaun Treweek:** conceptualization, methodology, validation, writing – review and editing, supervision, funding acquisition.

## Conflicts of Interest

The authors declare no conflicts of interest.

## Supporting information

Supplementary Material 1 Data extraction form.

Supplementary Material 2 SRQR Checklist.

Supplementary Material 3 Bibliography.

## Data Availability

Data sharing is not applicable to this article as no datasets were generated or analysed during the current study.
