# Supplementary material for: Do Funders, Regulators, and Ethics Bodies Support Informative Trials? A Content Analysis of Global Guidance Documents
Source: J Eval Clin Pract. 2026 Jan 22;32(1):e70356. doi: 10.1111/jep.70356 (PMC12826411; doi:10.1111/jep.70356)
Supplement: Supplementary file 1 — Supplementary Material 1 Data extraction form. [file JEP-32-0-s002.pdf]

Section 1: Document specifics	
Country of origin:	
Publisher:	
Document type:	
Document title:	
Year:	
URL (or other source):	

Section 2: Conditions for informativeness and relevant exemplar(s) as evidenced in the document			
**Conditions for informativeness:**	**Recommended process or action to improve informativeness (see: Prowse et al, 2024):**	**Supporting examples from the literature:**	**Additional notes:**
**1. Importance:** the trial hypothesis is likely to inform an important scientific, medical, or policy decision	☐ Implement a formal process of research priority or agenda setting that leverages existing structures and systems	Identification and prioritisation of research questions through evidence synthesis (including systematic reviews, scoping reviews, and other forms of evidence mapping), Value of Information (VOI) and other economic analyses and stakeholder consultations	
	☐ Develop fit-for-purpose (e.g., aligned with study intent) quality enhancement initiatives to support the early conceptualisation of research questions	Consider if current quality definitions and frameworks (e.g., INQUIRE) within trials research are aligned with the study intent and how quality enhancement initiatives may differ for low-resource settings	
	☐ Include patients as partners at the outset of the trial development process	Emphasis on the role of patients in guiding the development of trial hypotheses to better address issues important to patients and so that the proposed intervention is more likely to be seen by patients as acceptable	
	☐ Extend and formalise traditional research practices through Open Science (OS)	Pre-registering prospective projects on online platforms or submitting a registered report where hypotheses, experimental design, and analytic plans are specified prior to data collection	
	☐ Other example(s) as captured in the document:		
**2. Design:** the trial methods are likely to provide meaningful	☐ Select fit-for-purpose tools to further support a process of informed trial design	Trial designs can be further improved through the use of appropriate design tools (*see Table 3*) as well as end-to-end management platforms (e.g., from trial inception through	

evidence related to the study hypothesis			final reporting) and frameworks or other guiding documents intended to reduce research waste	
	☐	Establish research forums and other collaborative networks as a source of trial design feedback and support	Research forums and other collaborative networks can provide useful feedback when considering trial design and conduct such as obtaining approvals, data management, and developing good work relationships with funders; low-resource settings may also uniquely benefit from further collaboration when considering trial design and implementation	
	☐	If appropriate, consider pragmatic trial design approaches that evaluate the effectiveness of interventions under real-world conditions	Design tools such as PRECIS-2 and the GetReal Trial Tool can help conceptualise and inform both explanatory and/or pragmatic elements of trial design with the intent of creating trials that match design decisions to the intended decision-making purpose of the trial	
	☐	Integrate a patient-centric approach throughout the trial design process (e.g. weighing participant considerations such as convenience, risk to benefit ratio, social interaction, partnership, and altruism)	Consideration of patient needs, notably in early phase trial development, can lead to fewer protocol amendments, improved endpoints, improved feasibility (recruitment and retention), and higher patient satisfaction	
	☐	Consider a diversity of expertise within trial teams to ensure conditions for informativeness are incorporated throughout all aspects of trial planning and execution	Expertise should be sought across all areas of trial design and development including information technology, data development, scientific protocol development, clinical affairs, and financial operations; diversity is notably relevant to multicentre trials where expertise may span multiple teams working to achieve a shared outcome	
	☐	Seek further protocol guidance and feedback from anticipated trial site staff and team members	Site staff involvement in protocol development can further understanding of local context, capacity, and culture as well as ensuring that anticipated team members understand the protocol prior to trial initiation	
	☐	Use existing registry data to further inform clinical trial design	Potential applications of existing registry data include informing aspects of trial design such as sample size, or the use of additional tools to predict the early termination of a trial based on previous outcomes	

	☐	Further deliberation of critical ethical issues within trial protocol development	Substantive discussion of specific ethical issues is rarely included in clinical trial protocols and current reporting guidelines (e.g., SPIRIT, CONSORT) may not adequately support protocol writers, study teams, sponsors, ethics committees and reviewers in adequately addressing ethical issues	
	☐	Accountability from trial funders and/or sponsors to ensure trial design considerations are appropriate and adequately justified	Trial funders and/or sponsors should question the selection and validation of outcomes, interventions and comparators, sample sizes and suggested follow-up within a trial design; validation scales (e.g., COMET) may assist in choosing appropriate measures	
	☐	Utilise benefit-risk assessments for trials with more than one outcome of interest (e.g., superiority, equivalence, and non-inferiority trials)	Benefit-risk methodologies could be used to assess outcomes simultaneously and consider trade-offs while helping to ensure research hypotheses are answered appropriately (e.g., the effectiveness of the primary health outcome, relative to safety and cost)	
	☐	Conduct a scientific design review after the peer review process but prior to a funding commitment	Prior to trial funding, further consideration of the trial design, biostatistics, and research methods are needed to enhance understanding of trial informativeness and reduce research waste	
	☐	Improve the completeness of trial protocols by enhancing patient-reported outcome (PRO) data	The PRO content of trials is often suboptimal despite providing valuable evidence to inform shared decision making, labelling claims, clinical guidance and health policy; use of supporting guidelines (e.g. SPIRIT-PRO) can provide recommendations for items that should be included in protocols in which PROs are a primary or key secondary outcome	
	☐	Implement a ‘quality-by-design’ approach to clinical trials	A ‘quality-by-design’ approach enables organizations to prioritise the most critical determinants of a trial’s quality, identify non-essential activities that can be eliminated to streamline trial conduct and oversight, and formulate appropriate plans to define, avoid, mitigate, monitor, and address important errors	
	☐	Other example(s) as captured in the document:		

**3. Feasibility:** the trial must be demonstrably feasible (e.g., it must have a realistic plan for recruiting sufficient participants)	☐	Integrate qualitative evidence when assessing the feasibility of a trial	Qualitative research findings can further explore lived experiences of a disease condition, such as working within a specific healthcare system, and other barriers and enablers to participation within a trial	
	☐	If appropriate, consider a pilot or feasibility study to avoid research waste and de-risk funding investment(s)	Feasibility studies were found to be potentially useful in assessing whether a more expensive, large-scale trial was merited; feasibility studies of pragmatic trials may differ, and should consider feasibility objectives specifically relevant to areas of uncertainty for pragmatic trials	
	☐	Utilise the knowledge of local health professionals to better inform feasibility or pilot studies	Integration of local health professionals within the trial process can further contextualize issues of recruitment and retention, and help to ensure that the required sample size can be reached	
	☐	Include community members in participant recruitment and retention strategies	Concepts of community will vary by global context, but may be particularly relevant in low-resource settings when considering trial feasibility; community members can provide valuable feedback within retention strategies such as best practices for communication with potential trial participants	
	☐	Other example(s) as captured in the document:		
**4. Integrity**: the trial must be conducted and analysed in a scientifically valid manner that is faithful to the design	☐	Further external information about the treatment effect should be used to inform aspects of the trial analysis	Evidence synthesis of external resources can better inform aspects of trial analysis including further understanding of the treatment effect (e.g., through meta-analyses, or indirect comparisons), and reduction in bias in the presentation of analysis and trial results	
	☐	Further development of skills-based training to ensure the quality conduct of trials	Good clinical practice (GCP) describes the scientific and ethical considerations involved in the quality conduct of trials; trial teams may benefit from additional training beyond GCP certification in obtaining informed consent, ensuring protocol compliance, and protecting participants' health and safety	
	☐	Registration of Clinical Trial Units (CTUs) within wider national or international networks	Safeguards capacity for the development and delivery of high-quality trials by ensuring key competencies for conduct are met within a	

			CTU under the guidance of appropriate expertise	
	☐	Other example(s) as captured in the document:		
**5. Reporting:** systems are in place to ensure timely, complete, and accurate reporting	☐	Mandatory requirement to proactively register trials alongside a supporting system of monitoring to ensure compliance with registration, up-to-date record keeping, and timely publication of results	Publicly specifying details on trial methodology and conduct before enrolling participants increases transparency, decreases selective reporting and subsequent publication bias, and ensures an ethical responsibility to publicly report trial results	
	☐	Further reporting within appropriate registries	Patient registries and databases can further support clinical research and may be vital to assessing the feasibility of trials for areas such as rare or orphan diseases; reporting should consider the relevance of the trial to future research and contribute to registries as appropriate	
	☐	Reporting of health equity considerations	Transparency and completeness within the trial reporting process should consider how the presentation of the intervention and trial results may influence policy making and other decisions for those who are currently under-served by health research and health services	
	☐	Other example(s) as captured in the document:		

Section 3: Rubric to assess overall document informativeness		
**Select one:**	**Number of conditions for informativeness:**	**Descriptor:**
☐	All conditions for informativeness identified	Informativeness is **comprehensively described** with extensive detail and examples
☐	3-4 conditions for informativeness identified	Informativeness is **reasonably described**, but may benefit from further examples or elaboration
☐	1-2 conditions for informativeness identified	Informativeness is **partially described** with limited explanation
☐	No conditions for informativeness identified	Informativeness is **not described**
