# Supplementary material for: Do Funders, Regulators, and Ethics Bodies Support Informative Trials? A Content Analysis of Global Guidance Documents
Source: J Eval Clin Pract. 2026 Jan 22;32(1):e70356. doi: 10.1111/jep.70356 (PMC12826411; doi:10.1111/jep.70356)
Supplement: Supplementary file 3 — Supplementary Material 3 Bibliography. [file JEP-32-0-s001.pdf]

Supplementary Material 3:
Bibliography of Selected Documents by Document Type and Geography

Section 1: Funder documents

Australia

1. Australian Government National Health and Medical Research Council. *Competencies for Australian Academic Clinical Trialists*. May 2018. Accessed May 2, 2025. https://www.nhmrc.gov.au/sites/default/files/documents/attachments/competencies-for-australian-academic-clinical-trialists.pdf

---

Canada

2. Canadian Institutes of Health Research. *Applying CIHR's Research Excellence Framework – Best Practices for Clinical Trials*. June 2024. Accessed May 10, 2025. https://cihr-irsc.gc.ca/e/53947.html
3. Canadian Institutes of Health Research. *Project Grant Program: Application Process — RCT Evaluation Criteria and Headings*. January 2025. Accessed April 28, 2025. https://cihr-irsc.gc.ca/e/49806.html#a4

Germany

4. Deutsche Forschungsgemeinschaft (DFG, German Research Foundation). *Proposal Preparation Instructions: Clinical Trials Programme – Draft Proposals*. 2022. Accessed May 1, 2025. https://www.dfg.de/resource/blob/167630/46acfe961013fab4bb4896205ec2ed6a/17-03-en-data.pdf
5. Deutsche Forschungsgemeinschaft (DFG, German Research Foundation). *Guidelines: Clinical Trials Programme*. 2022. Accessed May 1, 2025. https://www.dfg.de/resource/blob/167624/cb5b268505e174d36269c4ff8829898e/17-01-en-data.pdf

---

India

6. Indian Council of Medical Research. *ICMR Policy on Research Integrity and Publication Ethics 2019*. 2019. Accessed April 28, 2025. https://www.icmr.gov.in/icmrobject/uploads/Guidelines/1724844182_icmr_pripe2019.pdf

---

Ireland

7. Health Research Board. *Clinical Trials and Interventions Research Governance Policy*. 2019. Accessed April 30, 2025. https://www.hrb.ie/wp-content/uploads/2024/05/HRB-Clinical-Trials-and-Interventions-Governance-Policy-1.pdf

---

Italy

8. Istituto Superiore di Sanità (ISS). *Promozione dell'integrità della ricerca: linee di indirizzo dell'Istituto Superiore di Sanità [To promote research integrity: guidelines of the Italian Istituto Superiore di Sanità]*. 2022. Accessed April 28, 2025. https://www.iss.it/documents/20126/0/Linee+vers+1+-+c.p..pdf/9925f595-1eac-a4ab-e231-51a62a1ccf2d?t=1675517579958

Multinational

9. European Commission (European Union). *Information on Clinical Studies*. 2025. Accessed May 10, 2025. https://ec.europa.eu/info/funding-tenders/opportunities/docs/2021-2027/horizon/temp-form/af/information-on-clinical-studies_he_en.docx

10. Wellcome. *Clinical Trials Policy*. August 2021. Accessed May 11, 2025. https://wellcome.org/research-funding/guidance/policies-grant-conditions/clinical-trials-policy

---

United Kingdom

11. National Institute for Health and Care Research. *HTA Programme Stage 2 Guidance Notes (REALMS)*. Version 2.5. March 2023. Accessed May 10, 2025. https://www.nihr.ac.uk/hta-programme-stage-2-guidance-notes-realms

12. Medical Research Council. *MRC Guidelines for Management of Global Health Trials: Involving Clinical or Public Health Interventions*. 2017. Accessed May 2, 2025. https://www.ukri.org/wp-content/uploads/2021/08/20220202_Guidelines-for-Global-Health-Trials-2017-v5-final.pdf

---

United States

13. National Institutes of Health. *Notice Number: NOT-OD-17-118*. 2017. Accessed April 28, 2025. https://grants.nih.gov/grants/guide/notice-files/NOT-OD-17-118.html

Section 2: Regulator documents

Australia

1. Australian Government, Department of Health and Aged Care Therapeutic Goods Administration. *Guidance on conducting clinical trials in Australia using "unapproved" therapeutic goods*. Last updated October 2024. Accessed May 12, 2025. https://www.tga.gov.au/resources/guidance/australian-clinical-trial-handbook

---

Brazil

2. Brazilian Health Regulatory Agency (ANVISA), Collegial Board of the Brazilian Health Surveillance Agency, ABRACRO (Brazilian Association of CROs). *Resolution RD No. 10, of February 20, 2015: Regulations for clinical trials with medical devices in Brazil*. 2015. Accessed April 28, 2025. https://cbdl.org.br/wp-content/uploads/2022/09/RDC_10_2015-regulation-for-clinical-trials-with-medical-devices-in-brazil-ABRACO.pdf

---

Ethiopia

3. Ethiopian Food & Drug Administration. *EFDA Clinical Trial Authorization Guideline EFDA/GDL/005*. April 2023. Accessed May 10, 2025. [.pdf available on request]

4. Ethiopian Food & Drug Authority. *Clinical Trial Protocol Template*. 26 August 2024. Accessed May 10, 2025. https://www.efda.gov.et/publication/clinical-trial-protocol-writing-template_versions-001/?lang=amh

---

India

5. Central Drugs Standard Control Organization, Directorate General of Health Services, Ministry of Health & Family Welfare, Government of India. *New Drugs & Clinical Trial Rules*. 2019. Accessed May 9, 2025. https://cdsco.gov.in/opencms/export/sites/CDSCO_WEB/Pdf-documents/NewDrugs_CTRules_2019.pdf

6. Indian Council of Medical Research. *Handbook for Applicants & Reviewers of Clinical Trials of New Drugs in India*. January 2017. Accessed May 9, 2025. https://jipmer.edu.in/sites/default/files/7.%20Hand%20Book%20for%20Applicants%20and%20Reviewers%20of%20Clinical%20Trials%20of%20New%20Drugs%20in%20India.pdf

---

Ireland

7. Health Products Regulatory Authority (HPRA). *Guide to Clinical Trials Conducted under the Clinical Trials Regulation (CTR) in Ireland*. 28 June 2023. Accessed May 1, 2025. http://hdl.handle.net/10147/641476

Kenya

8. Republic of Kenya; Ministry of Health, Pharmacy and Poisons Board. *Guidelines for the Conduct of Clinical Trials in Kenya*. September 2022. Accessed April 28, 2025. https://web.pharmacyboardkenya.org/download/guidelines-for-the-conduct-of-clinical-trials-in-kenya/?wpdmdl=4399&refresh=6762ac44858bb1734519876

Multinational

9. World Health Organization. *WHO guidance for best practices for clinical trials: Draft for public consultation*. 2023. Accessed April 28, 2025. https://cdn.who.int/media/docs/default-source/research-for-health/2023-07_who-guidance-for-best-practices-for-clinical-trials_draft-for-public-consultation.pdf?sfvrsn=7a5c9fa5_4

10. World Health Organization. *Strengthening clinical trials to provide high-quality evidence on health interventions and to improve research quality and coordination*. 27 May 2022. Accessed April 28, 2025. https://apps.who.int/gb/ebwha/pdf_files/WHA75/A75_R8-en.pdf

---

Nigeria

11. National Agency for Food & Drug Administration & Control (NAFDAC) Drug Evaluation & Research (DER) Directorate. *Guidelines for Clinical Trial Application in Nigeria 2024*. November 2024. Accessed April 28, 2025. https://nafdac.gov.ng/wp-content/uploads/Files/Resources/Guidelines/Clinical_Guidelines_2024/Guidelines-for-Clinical-Trial-Application-in-Nigeria.pdf

---

South Africa

12. Department of Health; Republic of South Africa. *South African Good Clinical Practice: Clinical Trial Guidelines*. 2020. Accessed April 28, 2025. https://www.sahpra.org.za/wp-content/uploads/2021/06/SA-GCP-2020_Final.pdf

---

United Kingdom

13. Medicines & Healthcare products Regulatory Agency (MHRA). *Guidance on legislation: Clinical investigation of medical devices – statistical considerations*. 2021. Accessed

May 10, 2025.
https://assets.publishing.service.gov.uk/media/62f529bb8fa8f50b57783ccf/Statistical_considerations_clinical_investigations_-_May_2021.pdf

---

United States

14. Food & Drug Administration. *Enhancing the Diversity of Clinical Trial Populations — Eligibility Criteria, Enrollment Practices, and Trial Designs Guidance for Industry*. 2020. Accessed April 28, 2025. https://www.fda.gov/regulatory-information/search-fda-guidance-documents/enhancing-diversity-clinical-trial-populations-eligibility-criteria-enrollment-practices-and-trial

Section 3: Ethics documents

Australia

1. National Health and Medical Research Council. *National Statement on Ethical Conduct in Human Research*. 2023. Accessed May 8, 2025. https://www.nhmrc.gov.au/about-us/publications/national-statement-ethical-conduct-human-research-2023

Canada

2. Government of Canada. *TCPS 2 (2022) – Chapter 11: Clinical Trials*. 2022. Accessed April 28, 2025. https://ethics.gc.ca/eng/tcps2-eptc2_2022_chapter11-chapitre11.html

---

Germany

3. Deutsche Forschungsgemeinschaft (DFG, German Research Foundation). *Guidelines for Safeguarding Good Research Practice: Code of Conduct*. April 2022 (Revised version 1.1). Accessed April 30, 2025. https://www.dfg.de/resource/blob/174052/1a235cb138c77e353789263b8730b1df/kodex-gwp-en-data.pdf

---

India

4. Indian Council of Medical Research. *National Ethical Guidelines for Biomedical and Health Research Involving Human Participants*. 2017. Accessed May 7, 2025. https://ethics.ncdirindia.org/asset/pdf/ICMR_National_Ethical_Guidelines.pdf

---

Kenya

5. Kenya Medical Research Institute. *Scientific Ethics Review Unit (SERU) Guidelines for Writing Project Proposals*. 2019. Accessed April 28, 2025. https://www.kemri.go.ke/wp-content/uploads/2019/11/KEMRI-Proposal-Format.pdf

---

Multinational

6. Wellcome. *Research Involving Human Participants Policy*. September 2021. Accessed April 28, 2025. https://wellcome.org/grant-funding/guidance/policies-grant-conditions/research-involving-human-participants-policy

---

Nigeria

7. Federal Ministry of Health. *National Code of Health Research Ethics*. 2007. Accessed April 28, 2025. https://www.nhrec.net/nhrec/NCHRE_July%2007.pdf

---

South Africa

8. National Department of Health. *South African Ethics in Health Research Guidelines: Principles, Processes and Structures*. 2024. Accessed May 8, 2025. https://www.health.gov.za/wp-content/uploads/2024/05/NDoH-2024-Health-Research-Guidelines-3rdEdition-v0.1.pdf

9. South African Medical Research Council Human Research Ethics Committee. *Ethics Committee Checklist for Quantitative Research*. 2022. Accessed May 8, 2025. https://www.samrc.ac.za/sites/default/files/attachments/2022-12/quantitativeresearch.docx

---

United Kingdom

10. National Health Service Health Research Authority. *Peer/Scientific Review of Research and the Role of NRES Research Ethics Committees (RECs)*. April 2012. Accessed May 7, 2025. https://s3.eu-west-2.amazonaws.com/www.hra.nhs.uk/media/documents/peer-scientific-review-of-research-and-the-role-of-nres-research-ethics-committees-.pdf
